# Age-related differences in the vascular function and structure of South Africans living with HIV

**DOI:** 10.4102/sajhivmed.23i1.1335

**Published:** 2022-02-24

**Authors:** Anisca Louwrens, Carla M.T. Fourie, Shani Botha-Le Roux, Yolandi Breet

**Affiliations:** 1 Hypertension in Africa Research Team (HART), School for Physiology, Nutrition and Consumer Science, Faculty of Health Sciences, North-West University, Potchefstroom, South Africa; 2 MRC Research Unit for Hypertension and Cardiovascular Disease, Faculty of Health Sciences, North-West University, Potchefstroom, South Africa

**Keywords:** arterial stiffness, carotid intima-media thickness, antiretroviral treatment, early vascular ageing, multi-morbidity

## Abstract

**Background:**

As the life expectancy of people living with the HIV increases because of antiretroviral treatment (ART), their risk for vascular co-morbidities and early vascular ageing (EVA) also increases.

**Objective:**

We aimed to investigate whether HIV infection relates to vascular structure and function in black South African adults and whether this relationship is age dependent.

**Method:**

This cross-sectional study carried out in urban and rural areas of North West province, South Africa, included 572 age- and sex-matched people living with HIV (PLWH) and without HIV. Participants from the EndoAfrica study and PURE study were stratified according to tertiles of age. Measures of vascular structure (carotid intima-media thickness) and function (carotid-femoral pulse wave velocity, central systolic blood pressure, central pulse pressure and pulse pressure amplification) were determined.

**Results:**

Blood pressure measures were lower in PLWH compared with their controls (all *P* ≤ 0.001), especially in the younger and middle-aged groups (all *P* ≤ 0.031), whilst vascular measures did not differ (all *P* ≥ 0.611). In multivariate linear regression analyses, vascular measures were not associated with a HIV- positive status in either the total or any of the age groups.

**Conclusion:**

Black South Africans living with HIV have a less adverse blood pressure profile than their counterparts without HIV. The HIV-positive status was not associated with measures of vascular structure or function in any age group. The results suggest that HIV does not contribute to EVA in this population; however, further longitudinal investigation is warranted.

## Introduction

The HIV epidemic is a global health problem with South Africa contributing a large number of people living with HIV (PLWH); of the 38 million PLWH worldwide, 7.7 million reside in South Africa.^1^ Although communicable diseases, including HIV, were the leading cause of death in 2012,^2^ the aetiology of mortality in sub-Saharan Africa (SSA) has shifted to a combination of communicable and non-communicable diseases since then.^3^ Approximately 69.4% of the South African population older than 40 years are battling multimorbidity, which is defined as two or more simultaneously existing chronic diseases.^4^ Multimorbidity adversely affects vascular health, causing acceleration in the vascular ageing process.^5,6,7^ Physiological, vascular ageing occurs with the progression of age and is a gradual and continuous process, characterised by structural and/or mechanical changes within the vessel wall.^8,9^ These mechanical and structural alterations that occur with vascular ageing may result in arterial stiffness and an increased carotid intima-media thickness (cIMT),^10^ which can affect some individuals prematurely, a term coined early vascular ageing (EVA).^11^ The prevalence of EVA causes an accelerated trajectory of prematurely developing hypertension, subclinical cardiovascular damage and CVD.^11^ An adverse transition from physiological vascular ageing to EVA occurs with exposure to risk factors, such as HIV,^12,13^ and factors commonly related to the disease, namely hypertension,^14^ dyslipidaemia,^15^ chronic low-grade inflammation^16^ and oxidative stress. Data on EVA in PLWH, especially in SSA, are scant as the majority of studies have been carried out on HIV-1 subtype B. However, a previous study carried out by our group found an indication of accelerated vascular ageing in older, never treated HIV-positive South Africans (aged > 50 years), as well as probable early atherosclerosis and endothelial dysfunction.^17^ Diabetes mellitus^18^ and dysglycaemia,^19^ as well as factors related to an unhealthy lifestyle,^20,21,22^ also lead to acceleration of the vascular ageing process.^23^ Vascular comorbidities, such as arterial stiffness and atherosclerosis,^5,6^ as well as cardiometabolic disturbances,^24,25^ become increasingly evident with the prolonged life expectancy of PLWH because of the effectiveness of antiretroviral treatment (ART).^26^ Hanna et al. indicated that the effect of HIV on the vasculature may differ through the lifespan of PLWH.^27^ HIV and/or ART-related vascular alterations may contribute to EVA.^8,9,10,28^ However, there is a lack of literature regarding the development of EVA in PLWH from South Africa, where the vast majority are infected with HIV type-1 subtype C phenotype.^29^ Therefore, we investigated whether HIV infection relates to vascular structure and function in South African adults on first-line ART at different ages.

## Research methods and design

### Setting and study population

This study includes data of participants residing in North West province of South Africa who participated in both the EndoAfrica study (vascular endothelial dysfunction: the putative interface of emerging cardiovascular risk factors affecting populations living with and without HIV in SSA) and the Prospective Urban and Rural Epidemiology (PURE) study. Data from the EndoAfrica study were collected from 2017 to 2018, and data collection for the PURE study took place in 2015. Both the studies were designed to determine the risk of developing cardiovascular disease amongst South Africans living with and without HIV. Both studies were previously described in more detail elsewhere.^30,31^

**FIGURE 1:**
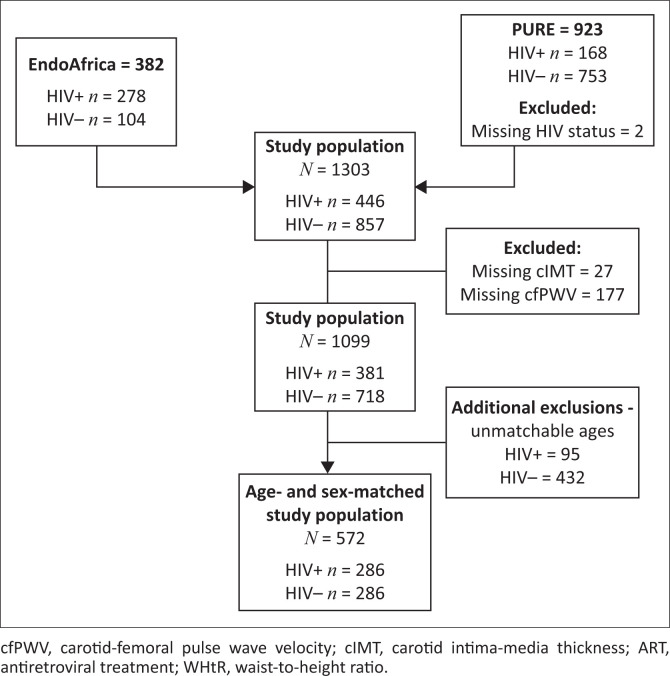
Study population of the EndoAfrica and the PURE studies.

The EndoAfrica study recruited participants from seven local clinics in and around Potchefstroom; the participants for the PURE study were recruited from urban and rural communities, which include Potchefstroom (urban), Ganyesa and Tlakgameng (rural). Participants in the EndoAfrica study were 18–60 years and in the PURE study were 42–88 years old. For this study, we included 286 PLWH and 286 HIV-free participants (control group). The participants in the group with HIV were matched for age and sex with those who were HIV free (Figure 1). The PLWH using treatment received first-line fixed dose combination ART.^26^ The exclusion criteria for both studies include PLWH using second- and third-line ART, individuals who refused HIV testing or to provide a blood sample, women who were pregnant or less than three months post-partum. Additional exclusions were made for missing data of the main variables and/or an unknown HIV status.

### Questionnaire and anthropometric measurements

A standardised questionnaire provided information regarding the age, sex, lifestyle patterns (which included tobacco use and alcohol consumption) and medication use in both studies.

Anthropometric measurements were performed according to the standardised methods of the International Society for the Advancement of Kinanthropometry.^32^ For both studies, height (stadiometer, SECA, Hamburg, Germany [EndoAfrica]; stadiometer, Leicester height measure, SECA, Birmingham, United Kingdom [UK] [PURE]), weight (flat scale, SECA Hamburg, Germany [EndoAfrica]; Precision Health Scale, A & D Company, Japan [PURE]) and waist circumference (WC) (lufkin steel anthropometric tape, W606PM, lufkin, Apex, United States [US] [EndoAfrica]; steel tape, lufkin, cooper tools, Apex NC, US [PURE]) were measured. Body mass index (BMI) was calculated according to international standards.

### Cardiovascular measurements

The brachial blood pressure, including systolic blood pressure (SBP), diastolic blood pressure (DBP), and heart rate of each seated participant were taken twice after 10 min of rest (OMRON M6 automatic digital blood pressure monitor, Omron Healthcare, Kyoto, Japan). The final measurement was used in subsequent analyses. We identified participants as hypertensive if SBP ≥ 140 mmHg and/or DBP ≥ 90 mmHg^33^ and/or if they used anti-hypertensive medication. Mean arterial pressure (MAP) was calculated, MAP = (SBP(2x*dbp*))/3.

Central SBP (cSBP), central pulse pressure (cPP) and carotid-femoral pulse wave velocity (cfPWV) were measured by arterial waveform analyses (SphygmoCor^®^ XCEL System, AtCor Medical Pty. Ltd, Sydney, Australia). The transit-distance method was used to measure cfPWV along the descending carotid-femoral artery. The 80% rule was applied for the calculation of the distance used.^34^ The cfPWV measurement was performed twice and repeated a third time if the two measurements differed by more than 3 m/s. The average was used for further analyses. Pulse pressure amplification (PPA) was defined as the ratio of the amplitude of the pulse pressure between a distal and proximal location.

Carotid intima-media thickness was measured in the PURE (SonoSite Micromaxx ultrasound system, SonoSite, Inc., Bothel, WA, US) and EndoAfrica (General Electric Vivid E9, GE Vingmed Ultrasound A/S, Horten, Norway) studies. Digitalised images were analysed with carotid vessel analyser automated software (Vascular Research Tools 6, Medical imageing applications, Coralville, Iowa, US) in the EndoAfrica study and with Artery Measurements Systems software (I version 1.139, Chalmers University of Technology, Gothenburg. Sweden) in the PURE study.

### Blood sampling and biochemical analyses

Blood samples were collected, centrifuged and aliquoted according to standardised methods, and were stored in −80 C bio-freezers for future analyses. Samples collected from the rural site during the PURE study were prepared at the on-site laboratory and immediately stored on dry ice (−18 °C) for a maximum of 5 days. These samples were then transported to the laboratory and stored at −80 °C.

In both studies, the Cobas Integra^®^ 400 Roche^®^ Clinical System (Roche Diagnostics, Indianapolis, IN, US) was used to determine glucose, total cholesterol (TC), high-density lipoprotein (HDL), low-density lipoprotein (LDL) and triglyceride levels using an enzymatic colourimetric method. High-sensitivity C-reactive protein (CRP) was analysed with the particle-enhanced turbidimetric assay method and gamma-glutamyl transferase (GGT) with the enzymatic colourimetric assay method.

Glycated haemoglobin (HbA1c) analyses were carried out with ethylenediamine tetraacetic acid whole blood samples in the EndoAfrica (turbidimetric inhibition immunoassay; Cobus Integra^®^ 400plus, Roche, Switzerland) and PURE (ion-exchange high-performance liquid chromatography method; D-10 Haemoglobin testing system from Bio-Rad #220-0101) studies.

In the EndoAfrica study, plasma samples were sent to the National Health Laboratory Services (NHLS) to determine the CD4+ cell count (Beckman COULTER^®^ EPICS^®^ XLTM Flow Cytometer, GMI Inc., Fullerton, CA, US). The finger-prick blood and a point-of-care device (PIMATM CD4, Alere, Jena, Germany) with the fixed volume cytometry analysis were used in the PURE study.

### HIV testing and counselling

Pre-counselling was given to each participant in both the EndoAfrica and PURE studies before HIV testing was performed with the first-response rapid HIV test (Premier Medical Corporation Limited, Daman, India). Positive HIV results were confirmed by the SD BOLINE HIV 1 / 2 3.0 (Standard Diagnostics, INC, Korea) rapid test in the EndoAfrica study and the ABON (Biopharm Corporation Limited Hanyzhou, China) in the PURE study.

### Statistical analyses

IBM^®^ SPSS^®^ Statistics, version 26.0 (IBM Corporation, Armonk, NY, USA) software was used for data analyses. In order to assess whether the data were normally distributed, we used graphical inspections (histograms and Q-Q plots) and numerically inspected the skewness and kurtosis of variables. Continuous data with a normal distribution are presented as arithmetic mean ± standard deviation and categorical data as proportions. For non-normally distributed data, we performed non-parametric tests and reported the median and interquartile ranges. The study population was stratified according to tertiles of age. The tertile ranges of the age groups for PLWH and without HIV, respectively, were as follows: younger ages were classified as 18–47 years versus 19–48 years, middle as 48–53 years versus 49–53 years, and older as 54–71 years versus 54–71 years. Differences between the groups were determined with independent T-tests or Mann-Whitney *U*-tests and Chi-square tests. Correlation analyses included Pearson or Spearman rank tests. In order to test for independent associations between the main outcome variables (cfPWV, PPA, cPP, cSBP and cIMT) and HIV infection, we performed multiple regression analyses (with the Enter method) in the total group, as well as stratified according to age tertile groups. All the models included HIV status, sex, BMI, TC, GGT, HbA1c, CRP, tobacco use, ART use and anti-hypertensive medication use. Mean arterial pressure was additionally included in the models, with cfPWV, cIMT and PPA as the dependent variables and heart rate in the model with PPA as the dependent variable. Adjustments for each dependent variable were made as follows: cfPWV (sex and MAP), cIMT (sex and MAP), cSBP (sex), cPP (sex) and PPA (sex, MAP, heart rate and height).

### Ethical considerations

This study complies with the requirements of the Declaration of Helsinki, and the Health Research Ethics Committee of the North-West University approved the EndoAfrica study (NWU-00045-15-A1), the PURE study (NWU-00016-10-A1), as well as this study (NWU-00367-20-A1). All procedures were explained to the participants before any measurements were made, and all participants gave written informed consent.

## Results

The characteristics of the study population are presented in Table 1. The PLWH and without HIV were matched for age (*P* = 0.098) and sex (*P* = 1.000), and their locality was comparable (*P* = 0.073). People living with HIV had a lower waist-to-height ratio (WtHR) and BMI (both *P* < 0.001) compared with the HIV-free group. With regard to cardiovascular measurements, all brachial blood pressures were lower in PLWH (all *P* < 0.049); however, no differences were found in either cfPWV, cIMT or PPA (all *P* ≥ 0.204). When comparing biochemical markers, GGT was higher in those with HIV (*P* < 0.001), whilst levels of glucose, glycated haemoglobin, TC, triglycerides and LDL-cholesterol (*P* ≤ 0.049) were lower. The CD4+ count was found to be 525.5 cells/µL for PLWH. Both tobacco use and alcohol consumption (*P* ≤ 0.014) were more frequent in PLWH.

**TABLE 1:** Characteristics of the study population (*N* = 286).

Characteristic	HIV+					HIV-					*P*
	*n*	%	Mean ± s.d.	Median	25th;75th percentiles	*n*	%	Mean ± s.d.	Median	25th;75th percentiles	
Women	194	67.8	-	-	-	194	67.8	-	-	-	1.000
Age (years)	-	-	48.66 ± 9.65	-	-		-	48.76 ± 9.73	-	-	0.098
Urban locality	219	76.6	-	-	-	199	69.6	-	-	-	0.073
**Anthropometry**											
WHtR		-	-	0.50	0.45;0.57	-	-	-	0.54	0.47;0.62	< **0.001**
Body mass index (kg/m^2^)		-	-	22.6	19.5;26.9	-	-	-	25.1	20.5;32.4	**< 0.001**
**Cardiovascular profile**											
Hypertensive	98	34.4	-	-	-	147	51.4	-	-	-	**< 0.001**
Brachial SBP (mmHg)	-	-	123 ± 23.3	-	-		-	128 ± 21.8	-	-	**0.013**
Brachial DBP (mmHg)	-	-	83 ± 13.0	-	-		-	85 ± 13.8	-	-	**0.049**
Brachial PP (mmHg)	-	-	40 ± 14.7	-	-		-	42 ± 13.1	-	-	**0.032**
Brachial MAP (mmHg)	-	-	96 ± 15.6	-	-		-	99 ± 15.7	-	-	**0.022**
Heart rate (beats/min)	-	-	72 ± 13.4	-	-		-	72 ± 14.3	-	-	0.599
Pulse pressure amplification‡	-	-	1.34 ± 0.13	-	-		-	1.33 ± 0.13	-	-	0.611
Central SBP (mmHg)	-	-	119 ± 18.4	-	-		-	124 ± 17.7	-	-	**0.001**
Central PP (mmHg)	-	-	36 ± 10.4	-	-		-	40 ± 10.6	-	-	**< 0.001**
Carotid-femoral PWV (m/s)†	-	-	8.17 ± 1.66	-	-		-	8.18 ± 1.75	-	-	0.204
Carotid IMT (mm)†	-	-	0.62 ± 0.11	-	-		-	0.63 ± 0.12	-	-	0.286
**Biochemical markers**											
Total cholesterol (mmol/L)	-	-	-	3.43	2.68;4.41	-	-	-	3.91	3.02;4.86	**< 0.001**
HDL-cholesterol (mmol/L)	-	-	-	1.14	0.86;1.46	-	-	-	1.19	0.88;1.64	0.105
LDL-cholesterol (mmol/L)	-	-	2.08 ± 0.91	-	-	-	-	2.31 ± 0.95	-	-	**0.004**
Triglycerides (mmol/L)	-	-	-	0.87	0.58;1.32	-	-	-	0.98	0.65;1.46	**0.049**
Glucose (mmol/L)	-	-	-	4.22	3.40;5.10	-	-	-	4.74	3.93;5.53	**< 0.001**
Glycated haemoglobin (%)	-	-	5.48 ± 0.61			-	-	5.91 ± 1.44	-		**< 0.001**
C-reactive protein (mg/L)	-	-	-	2.77	1.11;6.13	-	-	-	2.50	1.11;5.71	0.451
GGT (U/L)	-	-	-	47.1	26.0;110.5	-	-	-	30.0	16.9;63.7	**< 0.001**
CD4+ count (cells/µL)	-	-	525.5 ± 332.3	-	-	-	-	-	-	-	-
**Lifestyle variables**											
Tobacco use	143	50.5	-	-	-	113	39.9	-	-	-	**0.014**
Alcohol consumption	161	56.9	-	-	-	127	44.9	-	-	-	**0.005**
**Medication use**											
Antiretroviral therapy	213	76.3	-	-	-	-	-	-	-	-	-
Anti-hypertensive	44	15.7	-	-	-	72	25.5	-	-	-	**0.005**
Statins	4	1.4	-	-	-	10	3.5	-	-	-	0.174
Anti-glycaemic	1	0.4	-	-	-	18	6.4	-	-	-	**< 0.001**
Anti-inflammatory	8	2.8	-	-	-	17	6.0	-	-	-	0.100

SBP, systolic blood pressure; DBP, diastolic blood pressure; PP, pulse pressure; MAP, mean arterial pressure; PWV, pulse wave velocity; IMT, intima-media thickness; HDL, high-density lipoprotein; LDL, low-density lipoprotein; GGT, gamma-glutamyl transferase.

† , Adjusted for MAP;

‡ , adjusted for MAP, heart rate and height.

**FIGURE 2:**
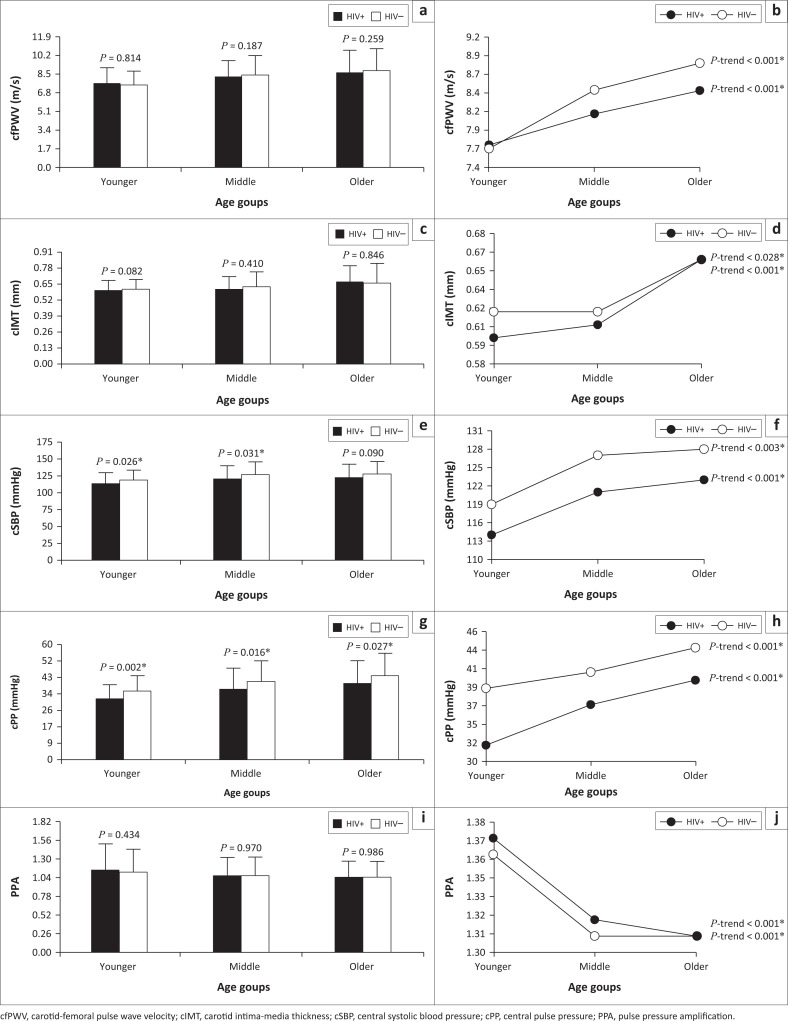
Adjusted differences in vascular measures (cfPWV, cIMT, cSBP, cPP and PPA) between people living with HIV (HIV+) and without HIV (HIV–) according to tertiles of age (on the left), as well as across different age tertile groups within the respective HIV status groups (on the right). Confounders included for each dependent variable: cfPWV (sex and mean arterial pressure), cIMT (sex and mean arterial pressure), cSBP (sex), cPP (sex) and PPA (sex, mean arterial pressure, heart rate and height).

In Figure 2 and Appendix Table 1-A1, we compared the adjusted differences in vascular measures (cfPWV, cIMT, cSBP, cPP and PPA) between the groups with and without HIV in different age tertiles (on the left), as well as across different age groups within the HIV and HIV-free groups, respectively (on the right). PLWH in the younger and middle age groups had lower cSBP (both *P* ≤ 0.031) and in all the age groups had lower cPP (all *P* ≤ 0.027) than those without HIV. Carotid-femoral pulse wave velocity, cIMT, cSBP and cPP increased, whilst PPA decreased across increased tertiles of age in both the HIV and HIV-free groups (all *P* ≤ 0.028).

**FIGURE 3:**
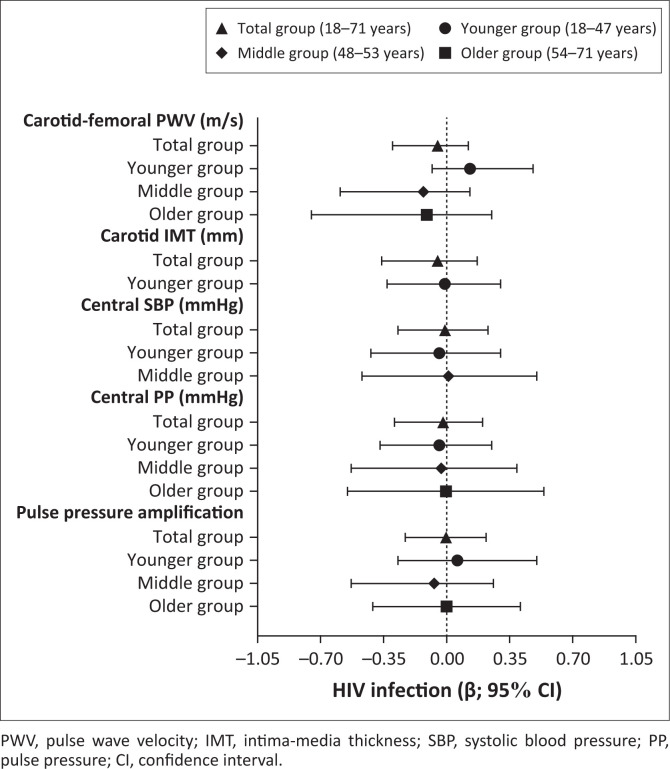
Associations between vascular measures and HIV status in the total group and in different age tertile groups. Models that were not significant (carotid IMT – middle and older group; central SBP – older group) were not included. Confounders included in all the models: HIV status, sex, body mass index, total cholesterol, gamma-glutamyl transferase, glycated haemoglobin, C-reactive protein, tobacco use, antiretroviral treatment use and anti-hypertensive medication use. Mean arterial pressure was additionally included in the models, with cfPWV, cIMT and PPA as the dependent variables. Heart rate was also included in the model, with PPA as the dependent variable.

We further determined whether vascular measures associated with the HIV status in the total group and in the respective age groups (Figure 3 and Appendix Table 2-A1), whilst taking cardiovascular risk factors and ART into account. No associations were observed between any of the vascular measures and HIV infection as the main independent variable (all *P* ≥ 0.162).

## Discussion

This study found that in a population of PLWH in South Africa, brachial blood pressure measurements (cSBP and cPP) were lower in younger and middle age groups and vascular markers (cfPWV, cIMT and PPA) were comparable with the HIV-uninfected population. In addition, we showed a lack of association between HIV infection and any of the vascular markers in any age group.

To the best of our knowledge, this was the first study to investigate age-related differences in the vascular structure and function between South Africans living with HIV and their age and sex-matched controls without HIV. We also determined whether an association between the HIV-positive status and these vascular measures exists within different age groups.

In addressing our first objective, we found that the group with HIV presented with less adverse central and brachial blood pressure profiles compared with their controls. In addition, the age-related deterioration of the vascular profile in the group with HIV was not as pronounced as expected, with cSBP being lower in the younger and middle age groups when compared with the group without HIV. Moreover, cPP was lower in PLWH in all the age groups when compared with their HIV-free counterparts. Data on the vascular profile of PLWH are controversial, and studies on this topic in a SSA context are scant. Previous studies both contradict^35,36,37,38^ and correspond^30,39,40,41^ to the study findings. Contrary to the study results, an Italian study in age-matched PLWH and individuals without HIV older than 18 years showed higher cSBP in those with HIV, which may have been attributed to renal damage.^35^ Msoka and colleagues concluded in a meta-analysis that cIMT, an early marker for atherosclerosis, was higher in PLWH compared with their controls in cross-sectional studies.^37^ The increased cIMT in the PLWH was associated with elevated levels of CRP and an adverse lipid profile.^37^ High CRP levels and an adverse lipid profile commonly occur in immunosuppressive states, such as HIV, where chronic low-grade systemic inflammation is known to prevail.^38^

Similar to our results, a narrative review of studies conducted in SSA concluded that treated PLWH had lower SBP and DBP, and hypertension compared with ART-naïve or individuals without HIV.^39^ We recently investigated the cardiovascular profile of PLWH and individuals without HIV in the EndoAfrica study, with a mean age of 42 years.^30^ The cardiovascular profile of these individuals included vascular markers similar to those in this current study, such as cfPWV, cIMT, cSBP and cPP.^30^ Neither the cardiovascular profile nor the CRP levels of those with HIV and participants without HIV in the EndoAfrica study differed,^30^ supporting the current findings that EVA was not evident in this study population with HIV. In another cross-sectional study, no increase in arterial stiffness, atherosclerosis or inflammation was found in PLWH, despite the presence of endothelial activation, when compared with ART-naïve or control groups.^41^

Considering the effect of ART on metabolic markers, it was reported that the lipid profile of Chinese PLWH who used Efavirenz-based ART was less adverse than those receiving Lopinavir or Ritonavir-based ART.^42^ The more favourable lipid profile of the group with HIV was also found in this South African study population using Efavirenz-based ART. Although there was no obvious effect on LDL-cholesterol in the Chinese study,^42^ this study found lower levels of LDL-cholesterol, TC and triglycerides in the HIV group (Table 1). This study’s less adverse glycaemic profile supports the rising notion that the cardiovascular profile of PLWH is less detrimental compared with their uninfected counterparts.^43^ These results support the findings of Shet et al., which showed better treatment outcomes for the HIV subtype C phenotype in South Africa, despite a higher baseline viral load, compared with other subtypes of HIV.^44^

Upon investigating whether an association exists between HIV infection and measures of vascular structure and function in different age groups, no significant results were found. In contrast with the study findings, a systematic review of studies conducted in SSA populations showed a positive association between HIV infection and atherosclerosis.^45^ It is known that atherosclerosis develops as a result of inflammation,^46^ and it is further suggested that the progression thereof is a plausible link to vasculopathy^47^ and EVA.^28,48^ However, Dillon et al. associated HIV infection with a lower SBP, DBP and BMI in a systematic review and meta-analysis on studies conducted in SSA.^40^ In our study population living with HIV, we found a lower cSBP and BMI compared with their counterparts without HIV in all the three age groups. However, we found no associations between cSBP and HIV infection in any of the age groups. The findings from previous studies, which found no association between HIV and arterial stiffness,^49^ hypertension^36^ or atherosclerosis,^50^ are in line with the study findings. Monteiro et al.^49^ reported a higher frequency of hypertension in the uninfected controls, which was also evident in this study.

As the carotid-femoral segment of the vasculature is highly sensitive to increases in blood pressure,^51^ the lower prevalence of hypertension in PLWH in this study could explain, at least in part, the absence of association of arterial stiffness with HIV status. Furthermore, PLWH using ART tend to develop cardiometabolic disturbances, resembling those found in the metabolic syndrome, such as lipodystrophy,^52^ dyslipidaemia^53^ and hyperglycaemia,^54^ none of which was encountered in the group with HIV of whom 76% received ART in this study.

The results on the association of HIV with vascular markers should be interpreted in light of the fact that we did not have ART adherence or viral load data. This cross-sectional study design limited us to infer cause and effect, and the study results need to be confirmed in a larger cohort. To the best of our knowledge, this was the first study to investigate the effect of age on the association between vascular structure and function and HIV infection in a South African population.

## Conclusion

The results of this study revealed that HIV-positive status did not associate with measures of vascular structure and function in any of the age groups. People living with HIV did not have a worse cardiovascular profile, and EVA was not evident in these people compared with those without HIV. The study findings warrant further longitudinal investigation that may observe individual vascular changes over time to determine whether South Africans living with HIV have an increased risk of developing CVD.

## Appendix 1

**TABLE 1-A1:** Characteristics of PLWH and without HIV, stratified by age tertile groups.

Variable	HIV+ (*n* = 98)						HIV- (*n* = 112)						*p*
	Mean ± s.d.	*Range*	*Median*	*25th;75th percentile*	*n*	%	Mean ± s.d.	*Range*	*Median*	*25th;75th percentile*	*n*	%	
**Younger group**													
Age range (years)	-	18–47	-	-	-	-	-	19–48	-	-	-	-	-
Women	-	-	-	-	63	64.3	-	-	-	-	71	63.4	1.000
Age (years)	38.6 ± 8.52	-	-	-	-	-	39.9 ± 8.83	-	-	-	-	-	0.294
Urban locality	-	-	-	-	92	93.9	-	-	-	-	80	71.4	< 0.001*
**Anthropometry**													
WHtR	-	-	0.49	0.43;0.56	-	-	-	-	0.53	0.45;0.60	-	-	0.043*
Body mass index (kg/m^2^)	-	-	22.8	19.6;26.3	-	-	-	-	24.3	20.5;31.9	-	-	0.021
**Cardiovascular profile**													
Hypertensive	-	-	-	-	28	28.6	-	-	-	-	44	39.3	0.111
Brachial SBP (mmHg)	117 ± 17.7	-	-	-	-	-	123 ± 17.0	-	-	-	-	-	0.020*
Brachial DBP (mmHg)	82 ± 11.9	-	-	-	-	-	85 ± 13.6	-	-	-	-	-	0.077
Brachial PP (mmHg)	35 ± 10.1	-	-	-	-	-	38 ± 9.73	-	-	-	-	-	0.073
Brachial MAP (mmHg)	94 ± 13.3	-	-	-	-	-	98 ± 14.1	-	-	-	-	-	0.040
Heart rate (beats/min)	72 ± 13.3	-	-	-	-	-	73 ± 15.2	-	-	-	-	-	0.597
Pulse pressure amplification‡	1.15 ± 0.36	-	-	-	-	-	1.12 ± 0.32	-	-	-	-	-	0.434
Central SBP (mmHg)	114 ± 15.7	-	-	-	-	-	119 ± 14.6	-	-	-	-	-	0.026*
Central PP (mmHg)	32 ± 7.16	-	-	-	-	-	36 ± 8.02	-	-	-	-	-	0.002*
Carotid-femoral PWV (m/s)†	7.67 ± 1.41	-	-	-	-	-	7.52 ± 1.26	-	-	-	-	-	0.814
Carotid IMT (mm)†	0.60 ± 0.08	-	-	-	-	-	0.61 ± 0.08	-	-	-	-	-	0.082
**Basic biochemical variables**													
Total cholesterol (mmol/L)	-	-	2.84	2.36;3.34	-	-	-	-	3.26	2.50;4.32	-	-	0.002*
HDL-cholesterol (mmol/L)	-	-	0.98	0.72;1.29	-	-	-	-	1.02	0.77;1.39	-	-	0.319
LDL-cholesterol (mmol/L)	1.65 ± 0.58	-	-	-	-	-	2.06 ± 0.85	-	-	-	-	-	< 0.001*
Triglycerides (mmol/L)	-	-	0.66	0.50;1.05	-	-	-	-	0.82	0.53;1.16	-	-	0.207
Glucose (mmol/L)	-	-	3.45	3.14;4.04	-	-	-	-	4.18	3.30;5.05	-	-	< 0.001*
Glycated haemoglobin (%)	-	-	-	-	-	-	5.92 ± 1.67	-	-	-	-	-	0.012*
C-reactive protein (mg/L)	-	-	1.88	0.53;5.33	-	-	-	-	1.80	0.71;4.87	-	-	0.836
GGT (U/L)	-	-	36.2	20.9;79.5	-	-	-	-	23.7	14.4;46.4	-	-	0.001*
CD4+ count (cells/µL)	492.6 ± 305.4	-	-	-	-	-	-	-	-	-	-	-	-
**Lifestyle variables**													
Tobacco use	-	-	-	-	48	49.5	-	-	-	-	40	36.7	0.068
Alcohol use	-	-	-	-	68	70.1	-	-	-	-	57	52.3	0.010*
**Medication use**													
Antiretroviral treatment	-	-	-	-	72	73.5	-	-	-	-	-	-	-
Anti-hypertensive	-	-	-	-	9	9.2	-	-	-	-	17	25.7	0.208
Statins	-	-	-	-	0	0.0	-	-	-	-	3	2.8	0.248
Anti-glycaemic	-	-	-	-	1	1.0	-	-	-	-	6	5.6	0.122
Anti-inflammatory	-	-	-	-	0	0.0	-	-	-	-	3	2.8	0.248
**Middle group**													
Age range (years)	-	48–53	-	-	-	-	-	49–53	-	-	-	-	-
Women	-	-	-	-	75	70.8	-	-	-	-	66	72.5	0.874
Age (years)	50.5 ± 1.80	-	-	-	-	-	51.0 ± 1.46	-	-	-	-	-	0.034*
Urban locality	-	-	-	-	77	72.6	-	-	-	-	56	61.5	0.127
**Anthropometry**													
WHtR	-	-	0.52	0.46;0.59	-	-	-	-	0.56	0.47;0.65	-	-	0.054
Body mass index (kg/m^2^)	-	-	22.9	19.8;28.6	-	-	-	-	26.1	20.6;33.7	-	-	0.015*
**Cardiovascular profile**													
Hypertensive	-	-	-	-	38	35.8	-	-	-	-	50	54.9	0.010*
Brachial SBP (mmHg)	125 ± 24.8	-	-	-	-	-	130 ± 23.9	-	-	-	-	-	0.113
Brachial DBP (mmHg)	84 ± 13.9	-	-	-	-	-	86 ± 13.3	-	-	-	-	-	0.340
Brachial PP (mmHg)	41 ± 14.4	-	-	-	-	-	44 ± 13.8	-	-	-	-	-	0.070
Brachial MAP (mmHg)	98 ± 16.9	-	-	-	-	-	101 ± 16.3	-	-	-	-	-	0.197
Heart rate (beats/min)	72 ± 14.0	-	-	-	-	-	73 ± 13.3	-	-	-	-	-	0.714
Pulse pressure amplification‡	1.07 ± 0.25	-	-	-	-	-	1.07 ± 0.26	-	-	-	-	-	0.970
Central SBP (mmHg)	121 ± 19.1	-	-	-	-	-	127 ± 19.0	-	-	-	-	-	0.031*
Central PP (mmHg)	37 ± 10.8	-	-	-	-	-	41 ± 10.8	-	-	-	-	-	0.016*
Carotid-femoral PWV (m/s)†	8.27 ± 1.45	-	-	-	-	-	8.43 ± 1.77	-	-	-	-	-	0.187
Carotid IMT (mm)†	0.61 ± 0.10	-	-	-	-	-	0.63 ± 0.12	-	-	-	-	-	0.410
**Basic biochemical variables**													
Total cholesterol (mmol/L)	-	-	3.71	2.87;4.85	-	-	-	-	4.12	3.44;4.83	-	-	0.074
HDL-cholesterol (mmol/L)	-	-	1.16	1.00;1.41	-	-	-	-	1.36	1.04;1.71	-	-	0.015*
LDL-cholesterol (mmol/L)	2.23 ± 1.02	-	-	-	-	-	2.35 ± 0.96	-	-		-	-	0.391
Triglycerides (mmol/L)	-	-	1.09	0.69;1.58	-	-	-	-	1.09	0.80;1.52	-	-	0.578
Glucose (mmol/L)	-	-	4.45	3.54;5.26	-	-	-	-	5.03	4.45;5.63	-	-	0.001*
Glycated haemoglobin (%)	5.50 ± 0.58	-	-	-	-	-	5.81 ± 1.04	-	-	-	-	-	0.011*
C-reactive protein (mg/L)	-	-	3.17	1.22;7.15	-	-	-	-	2.84	1.47;6.10	-	-	0.803
GGT (U/L)	-	-	55.0	32.9;130.8	-	-	-	-	37.1	18.3;77.1	-	-	0.006*
CD4+ count (cells/µL)	550.0 ± 386.7	-	-	-	-	-	-	-	-	-	-	-	-
**Lifestyle variables**													
Tobacco use	-	-	-	-	53	50.5	-	-	-	-	43	47.3	0.670
Alcohol use	-	-	-	-	55	52.4	-	-	-	-	39	42.9	0.199
**Medication use**													
Antiretroviral treatment	-	-	-	-	83	79.8	-	-	-	-	-	-	-
Anti-hypertensive	-	-	-	-	17	16.5	-	-	-	-	21	23.1	0.280
Statins	-	-	-	-	0	0.0	-	-	-	-	1	1.1	0.469
Anti-glycaemic	-	-	-	-	0	0.0	-	-	-	-	6	1.1	0.010*
Anti-inflammatory	-	-	-	-	2	1.9	-	-	-	-	7	1.1	0.086
**Older group**													
Age range (years)	-	54–71	-	-	-	-	-	54–71	-	-	-	-	-
Women	-	-	-	-	56	68.3	-	-	-	-	57	68.7	1.000
Age (years)	58.3 ± 3.99	-	-	-	-	-	58.3 ± 3.97	-	-	-	-	-	0.989
Urban locality	-	-	-	-	50	61.0	-	-	-	-	63	75.9	0.045*
**Anthropometry**													
WHtR	-	-	0.50	0.45;0.57	-	-	-	-	0.54	0.49;0.62	-	-	0.004*
Body mass index (kg/m^2^)	-	-	22.1	18.9;26.3	-	-	-	-	24.6	20.5;31.1	-	-	0.004*
**Cardiovascular profile**													
Hypertensive	-	-	-	-	32	39.5	-	-	-	-	53	63.9	0.003*
Brachial SBP (mmHg)	129 ± 25.4	-	-	-	-	-	133 ± 23.8	-	-	-	-	-	0.331
Brachial DBP (mmHg)	83 ± 12.9	-	-	-	-	-	85 ± 14.6	-	-	-	-	-	0.424
Brachial PP (mmHg)	46 ± 17.3	-	-	-	-	-	48 ± 13.8	-	-	-	-	-	0.409
Brachial MAP (mmHg)	98 ± 16.1	-	-	-	-	-	101 ± 16.9	-	-	-	-	-	0.366
Heart rate (beats/min)	71 ± 12.9	-	-	-	-	-	71 ± 14.2	-	-	-	-	-	0.970
Pulse pressure amplification‡	1.05 ± 0.22	-	-	-	-	-	1.05 ± 0.22	-	-	-	-	-	0.986
Central SBP (mmHg)	123 ± 19.3	-	-	-	-	-	128 ± 18.4	-	-	-	-	-	0.090
Central PP (mmHg)	40 ± 11.7	-	-	-	-	-	44 ± 11.7	-	-	-	-	-	0.027*
Carotid-femoral PWV (m/s)†	8.65 ± 2.02	-	-	-	-	-	8.83 ± 1.99	-	-	-	-	-	0.259
Carotid IMT (mm)†	0.67 ± 0.13	-	-	-	-	-	0.66 ± 0.16	-	-	-	-	-	0.846
**Basic biochemical variables**													
Total cholesterol (mmol/L)	-	-	4.18	3.42;4.88	-	-	-	-	4.68	3.65;5.66	-	-	0.038*
HDL-cholesterol (mmol/L)	-	-	1.30	0.93;1.67	-	-	-	-	1.23	1.02;1.64	-	-	0.976
LDL-cholesterol (mmol/L)	2.43 ± 0.46	-	-		-	-	2.60 ± 0.97	-	-		-	-	0.267
Triglycerides (mmol/L)	-	-	0.99	0.66;1.25	-	-	-	-	1.07	0.76;1.64	-	-	0.030*
Glucose (mmol/L)	-	-	4.87	4.23;5.36	-	-	-	-	5.03	4.49;5.60	-	-	0.143
Glycated haemoglobin (%)	5.49 ± 0.46	-	-		-	-	6.02 ± 1.49	-	-	-	-	-	0.004*
C-reactive protein (mg/L)	-	-	3.51	1.86;6.61	-	-	-	-	2.92	1.21;6.27	-	-	0.281
GGT (U/L)	-	-	49.0	27.8;100.3	-	-	-	-	36.8	20.7;76.1	-	-	0.080
CD4+ count (cells/µL)	533.9 ± 281.2	-	-	-	-	-	-	-	-	-	-	-	-
**Lifestyle variables**													
Tobacco use	-	-	-	-	42	51.9	-	-	-	-	30	36.1	0.059
Alcohol use	-	-	-	-	38	46.9	-	-	-	-	31	37.3	0.268
**Medication use**													
Antiretroviral treatment	-	-	-	-	58	75.3	-	-	-	-	-	-	-
Anti-hypertensive	-	-	-	-	18	22.5	-	-	-	-	34	41.0	0.012*
Statins	-	-	-	-	4	5.0	-	-	-	-	6	7.2	0.746
Anti-glycaemic	-	-	-	-	0	0.0	-	-	-	-	6	7.2	0.029*
Anti-inflammatory	-	-	-	-	6	7.5	-	-	-	-	7	8.4	1.000

Note: Data are expressed as arithmetic mean ± standard deviation; median (25th and 75th percentiles) or percentage of *N*.

* , *P*-values of ≤ 0.050 are regarded as statistically significant.

† , Adjusted for mean arterial pressure;

‡ , Adjusted for mean arterial pressure, heart rate and height.

WHtR,waist-to-height ratio; SBP, systolic blood pressure DBP, diastolic blood pressure; PP, pulse pressure; MAP, mean arterial pressure; PWV, pulse wave velocity; IMT, intima-media thickness; HDL, high-density lipoprotein; LDL, low-density lipoprotein; GGT, gamma-glutamyl transferase.

**TABLE 2-A1:** Multiple regression analyses between vascular measures and HIV status in the total group and in different age tertile groups.

Variable	Total group (≥ 18 ≤ 71) (*N* = 572)			Younger group (≥ 18 ≤ 47) (*n* = 193)			Middle group (≥ 48 ≤ 53) (*n* = 214)			Older group (≥ 54 ≤ 71) (*n* = 165)		
	β	95% CI	*P*	β	95% CI	*P*	β	95% CI	*P*	β	95% CI	*P*
**Carotid-femoral pulse wave velocity (m/s)**												
Adjusted *R*²	0.415	-	-	0.440	-	-	0.367	-	-	0.299	-	-
HIV status, infected	−0.05	−0.30	0.12	0.13	−0.08	0.48	−0.13	−0.59	0.13	−0.11	−0.75	0.25
Age (years)	0.27	0.19	0.35**	-	-	-	-	-	-	-	-	-
Sex, men	0.09	0.02	0.35*	0.17	0.05	0.50*	0.14	0.003	0.55*	−0.01	−0.43	0.38
BMI (kg/m^2^)	−0.19	−0.27	−0.11**	−0.24	−0.32	−0.09**	−0.14	−0.25	< 0.001	−0.19	−0.42	−0.04*
MAP (mmHg)	0.45	0.38	0.52**	0.48	0.32	0.54**	0.51	0.35	0.56**	0.52	0.42	0.74**
TC (mmol/L)	−0.09	−0.17	−0.01*	0.03	−0.08	0.13	−0.11	−0.23	0.02	−0.08	−0.31	0.10
GGT (U/L)	0.15	0.07	0.22**	0.10	−0.10	0.11	0.22	0.09	0.31**	0.18	0.04	0.41*
HbA1c (%)	0.08	0.01;	0.15*	0.30	0.12	0.30**	0.05	−0.08	0.18	−0.03	−0.21	0.15
CRP (mg/L)	−0.03	−0.10	0.04	−0.002	−0.13	0.12	−0.001	−0.09	0.09	−0.05	−0.25	0.12
Tobacco use, yes	0.05	−0.05	0.24	0.15	0.04	0.45*	0.004	−0.23	0.24	0.05	−0.22	0.47
ART use, yes	−0.03	−0.27	0.16	−0.09	−0.44	0.14	0.05	−0.28	0.46	−0.03	−0.58	0.44
Anti-HT medication use, yes	0.06	−0.02	0.33	0.06	−0.17	0.43	0.08	−0.09	0.46	0.04	−0.29	0.47
**Carotid intima-media thickness (mm)**												
Adjusted *R*²	0.037	-	-	0.075	-	-	0.008	-	-	0.036	-	-
HIV status, infected	−0.05	−0.36	0.17	−0.12	−0.33	0.30	NS	-	-	NS	-	-
Age (years)	0.22	0.12	0.31**	-	-	-	-	-	-	-	-	-
Sex, men	0.06	−0.09	0.33	0.08	−0.14	0.36	NS	-	-	NS	-	-
BMI (kg/m^2^)	0.12	0.02	0.22*	0.11	−0.05	0.21	NS	-	-	NS	-	-
MAP (mmHg)	0.06	−0.03	0.15	0.11	−0.03	0.21	NS	-	-	NS	-	-
TC (mmol/L)	−0.02	−0.12	0.08	0.18	0.02	0.26*	NS	-	-	NS	-	-
GGT (U/L)	−0.03	−0.13	0.06	−0.09	−0.18	0.06	NS	-	-	NS	-	-
HbA1c (%)	0.05	−0.04	0.14	0.08	−0.05	0.14	NS	-	-	NS	-	-
CRP (mg/L)	−0.02	−0.10	0.07	0.09	−0.06	0.22	NS	-	-	NS	-	-
Tobacco use, yes	0.06	−0.07	0.29	−0.07	−0.32	0.13	NS	-	-	NS	-	-
ART use, yes	0.06	−0.14	0.40	0.05	−0.25	0.39	NS	-	-	NS	-	-
Anti-HT medication, yes	0.08	−0.02	0.42	0.06	−0.20	0.46	NS	-	-	NS	-	-
**Central systolic blood pressure (mmHg)**												
Adjusted *R*²	0.167	-	-	0.184	-	-	0.104	-	-	0.054	-	-
HIV status, infected	−0.01	−0.27	0.23	−0.04	−0.42	0.30	0.01	−0.47	0.50	NS	-	-
Age (years)	0.25	0.16	0.34**	-	-	-	-	-	-	-	-	-
Sex, men	0.04	−0.11	0.29	0.14	−0.04	0.54	−0.03	−0.45	0.29	NS	-	-
BMI (kg/m^2^)	0.23	0.14	0.33**	0.25	0.08	0.37*	0.24	0.07	0.40*	NS	-	-
TC (mmol/L)	−0.06	−0.15	0.04	−0.02	−0.16	0.12	0.07	−0.10	0.24	NS	-	-
GGT (U/L)	0.18	0.09	0.27**	0.24	0.08	0.34*	0.13	−0.02	0.29	NS	-	-
HbA1c (%)	0.06	−0.03	0.14	0.06	−0.07	0.16	0.12	−0.03	0.31	NS	-	-
CRP (mg/L)	−0.11	−0.19	−0.03*	−0.02	−0.18	0.14	−0.18	−0.72	0.04*	NS	-	-
Tobacco use, yes	0.03	-	-	0.09	−0.12	0.41	0.07	−0.17	0.46	NS	-	-
ART use, yes	−0.12	−0.12	0.22	−0.16	−0.66	0.08	−0.08	−0.66	0.32	NS	-	-
Anti-HT medication, yes	0.09	0.01	0.42*	0.26	0.30	1.04**	0.05	−0.23	0.51	NS	-	-
**Central pulse pressure (mmHg)**												
Adjusted *R*²	0.201	-	-	0.179	-	-	0.135	-	-	0.062	-	-
HIV status, infected	−0.02	−0.29	0.20	−0.04	−0.37	0.25	−0.03	−0.53	0.39	−0.003	−0.55	0.54
Age (years)	0.31	0.22	0.40**	-	-	-	-	-	-	-	-	-
Sex, men	0.02	−0.15	0.24	0.14	−0.03	0.46	−0.08	−0.53	0.17	0.12	−0.16	0.71
BMI (kg/m^2^)	0.247	0.18	0.36**	0.37	0.15	0.40**	0.28	0.11	0.42**	0.25	0.07	0.49*
TC (mmol/L)	0.02	−0.08	0.11	0.07	−0.06	0.17	0.12	−0.02	0.29	−0.14	−0.39	0.05
GGT (U/L)	−0.01	−0.10	0.08	0.02	−0.10	0.13	−0.04	−0.18	0.11	0.04	−0.39	0.05
HbA1c (%)	0.03	−0.06	0.11	0.10	−0.03	0.16	0.05	−0.10	0.23	0.03	−0.16	0.22
CRP (mg/L)	−0.13	−0.20;	−0.05*	−0.17	−0.29	−0.02*	−0.15	−0.24	−0.01*	−0.07	−0.29	0.11
Tobacco use, yes	0.03	−0.11	0.23	0.10	−0.08	0.36	0.07	−0.16	0.44	−0.05	−0.47	0.27
ART use, yes	−0.10	−0.45	0.05	−0.08	−0.43	0.20	−0.04	−0.55	0.38	−0.14	−0.87	0.24
Anti-HT medication, yes	0.05	−0.45	0.05	0.20	0.11	0.75*	−0.01	−0.37	0.33	0.07	−0.26	0.57
**Pulse pressure amplification**												
Adjusted *R*²	0.334	-	-	0.207	-	-	0.414	-	-	0.258	-	-
HIV status, infected	−0.004	−0.23	0.22	0.06	−0.27	0.50	−0.07	−0.23	0.27	<0.001	−0.41	0.41
Age (years)	−0.27	−0.36	−0.19**	-	-	-	-	-	-	-	-	-
Sex	0.17	0.18	0.54**	0.07	−0.19	0.44	0.19	0.14	0.75*	0.15	−0.03	0.65
BMI (kg/m^2^)	−0.03	−0.12	0.05	−0.09	−0.24	0.08	0.08	−0.06	0.21	−0.10	−0.26	0.06
MAP (mmHg)	0.01	−0.06	0.09	−0.21	−0.37	0.07*	0.07	−0.04	0.19	0.05	−0.26	0.06
Heart rate (bpm)	0.48	0.41	0.56**	0.42	0.26	0.53**	0.57	0.45	0.68**	0.43	0.26	0.57**
TC (mmol/L)	−0.05	−0.13	0.04	−0.05	−0.19	0.10	−0.12	−0.27	0.004	0.01	−0.15	0.18
GGT (U/L)	0.03	−0.06	0.11	−0.12	−0.26	0.04	0.11	−0.02	0.24	0.14	−0.03	0.30
HbA1c (%)	−0.03	−0.10	0.05	−0.10	−0.20	0.04	−0.03	−0.18	0.24	0.03	−0.12	0.17
CRP (mg/L)	0.06	−0.02	0.13	0.12	−0.03	0.31	0.05	−0.05	0.14	0.06	−0.09	0.23
Tobacco use, yes	0.03	−0.09	0.22	−0.02	−0.33	0.24	0.08	−0.10	0.41	0.04	−0.20	0.36
ART use, yes	0.01	−0.20	0.26	−0.13	−0.64	0.15	0.09	−0.21	0.59	0.03	−0.37	0.48
Anti-HT medication, yes	0.02	−0.14	0.24	−0.10	−0.68	0.14	−0.01	−0.33	0.27	0.13	−0.07	0.57

CRP, C-reactive protein; BMI, body mass index; MAP, mean arterial pressure; TC, total cholesterol; HbA1c, glycated haemoglobin; GGT, gamma-glutamyl transferase; ART, antiretroviral treatment; Anti-HT, anti-hypertensive.

* , *p* < 0.05;

** , *p* ≤ 0.001.
